# Long-Term Clinical and Molecular Changes in Dry Eye Disease and Chronic Ocular Pain

**DOI:** 10.3390/ijms26188918

**Published:** 2025-09-12

**Authors:** Cristina Valencia-Sandonís, Andrés Ángel Calderón-García, Marta Blanco-Vázquez, Laura Valencia-Nieto, Andrea Novo-Diez, Amanda Vázquez, Margarita Calonge, María J. González-García, Amalia Enríquez-de-Salamanca

**Affiliations:** 1 Institute of Applied Ophthalmobiology (IOBA), Universidad de Valladolid (UVa), Campus Miguel Delibes, Paseo de Belén 17, 47011 Valladolid, Spain; cristina.valencia@uva.es (C.V.-S.); andresangel.calderon@uva.es (A.Á.C.-G.); marta.blanco.vazquez@uva.es (M.B.-V.); laura.valencia@uva.es (L.V.-N.); andrea.novo@uva.es (A.N.-D.); amanda.vazquez@uva.es (A.V.); mcalonge@uva.es (M.C.); amalia.enriquez-salamanca@uva.es (A.E.-d.-S.); 2 Pain Unit, Alliance of University Hospitals, Health System of Castilla y León, Calle Dulzaina 2, 47012 Valladolid, Spain; 3 Biomedical Research Networking Center in Bioengineering, Biomaterials and Nanomedicine (CIBER-BBN), Campus Miguel Delibes, Paseo de Belén 17, 47011 Valladolid, Spain

**Keywords:** dry eye, chronic ocular pain, tear biomarkers, gene expression, miRNAs expression

## Abstract

Dry eye disease (DED) is a prevalent condition characterized by ocular surface inflammation and pain. This study evaluated the long-term progression of DED by analyzing clinical and molecular status, considering the impact of chronic ocular pain. Patients with DED were evaluated at two visits (V1 and V2) separated by at least two years. Evaluations included validated symptom questionnaires alongside slit-lamp examination, corneal sensitivity testing, and sub-basal nerve plexus analysis. Basal tear samples were collected for multiplex quantification of 20 cytokines and substance P (SP), and conjunctival cells were obtained to analyze 25 genes and 12 microRNAs (miRNA). Based on the presence or absence of chronic ocular pain, patients were then divided into two groups. Patients improved in DED-related symptoms, with no changes observed in ocular surface signs. Corneal dendritic cell density decreased, along with epidermal growth factor (EGF), fractalkine, and monocyte chemoattractant protein (MCP-1) tear levels, whereas interleukin (IL)-10 and SP tear levels increased. Neurotrophic tyrosine kinase, receptor, type *(NTRK)1* gene expression was significantly downregulated, especially in patients without chronic ocular pain. miR-665 expression decreased significantly in DED patients. Monitoring corneal dendritic cells, tear cytokines, and gene/miRNA expression offers promising tools for tracking DED progression. Distinguishing the presence of chronic ocular pain as a separate symptom is crucial to optimizing therapeutic strategies and DED progression.

## 1. Introduction

Dry eye disease (DED) is a common, complex, multifactorial, and symptomatic disease that significantly affects patient’s quality of life [1]. Its prevalence varies depending on the diagnostic criteria, disease severity, and population characteristics, with estimates ranging from 2.7% in individuals aged 20–29 to over 30% in women above 80 [2,3]. In Spain, a recent population-based survey reported a 12.3% prevalence of clinically diagnosed DED, with a notable proportion of young women (18–29 years old) reporting symptoms [4]. Several risk factors have been associated with DED, including increasing age, female sex, ocular surgeries, prolonged screen exposure, and mental health disorders such as anxiety and depression [2,3]. Given its high prevalence and clinical impact, DED represents a major public health issue and a significant burden on healthcare systems worldwide.

Pain is a fundamental protective mechanism that signals actual or potential tissue damage. In DED, tear film instability and hyperosmolarity activate corneal nociceptors, triggering nociceptive signaling through afferent pathways to the central nervous system. Although initially protective, sustained ocular surface stress promotes the release of inflammatory mediators and structural damage to nerve terminals, resulting in peripheral sensitization. If the noxious stimulus or inflammation persists, central sensitization may develop, amplifying pain responses at the level of the central nervous system [5,6].

Traditionally regarded as a symptom of DED, ocular pain is now increasingly recognized as a distinct closely interconnected clinical entity that may coexist with DED, complicating diagnosis and management [1,6]. These interrelated conditions are often difficult to differentiate, but accurate diagnosis is essential to guide effective treatment. Patients with chronic ocular pain secondary to DED may show poor response to conventional therapies and often require a multimodal approach that includes interventions targeting the nervous system [1,7].

In this context, the identification of biomarkers for diagnosing and monitoring DED and ocular pain is being extensively investigated, supported by the accessibility and minimally invasive nature of tear fluid and conjunctival cell sampling [8,9]. Elevated levels of cytokines and neuropeptides involved in inflammation and/or pain have been detected in the tears of patients with DED. Cytokines such as interleukin (IL)-1 receptor antagonist (Ra), IL-6, IL-8/CXCL8, and matrix metalloproteinase (MMP)-9 have been associated with DED [9,10,11]. Previous studies have identified an association between the concentration or percentage of detection levels of IL-9 in tears and the development of chronic ocular pain; similarly, associations have been published between ocular surface inflammation and IL-2, IL-8/CXCL8, macrophage inflammatory protein (MIP)-1α/CCL3, and interferon (IFN)-γ [9].

Moreover, the study of gene and microRNA (miRNA) expression related to inflammation and pain expression is emerging as a promising tool in ocular surface disease research. The analysis of conjunctival gene expression has gained attention as a valuable approach for diagnosing and monitoring inflammatory ocular surface diseases [12,13,14]. miRNAs are short noncoding RNA molecules that regulate gene expression at the post-transcriptional level which have been proposed as very promising biomarkers because of their high stability. Emerging evidence highlights a reciprocal relationship between miRNAs and epigenetic regulation in which miRNAs influence the expression of epigenetic regulators while their own transcription is subject to epigenetic control [15]. Changes in miRNA expression have also been actively investigated in the context of ocular surface inflammation [16,17,18,19]. In patients with DED, upregulation of inflammation-related miRNAs has been reported, including miR-127-5p, miR-1273h-3p, miR-1288-5p, miR-130b-5p, miR-139-3p, miR-1910-5p, miR-203b-5p, miR-22-5p, and miR-4632-3p [1,19]. Animal models of Sjögren’s syndrome have also demonstrated altered tear miRNA profiles associated with autoimmune-mediated DED [18]. In particular, miRNA-146a-5p acts as a regulator of inflammation in DED by inhibiting target genes such as *IRAK1*, which leads to decreased expression of proinflammatory mediators including IL-6 and tumor necrosis factor (TNF)-α [17].

Thus, the aim of this study was to evaluate the long-term progression of DED by assessing clinical and molecular changes over a minimum follow-up period of two years. Specifically, we analyzed the evolution of symptoms, clinical signs, tear levels of cytokines and neuropeptides related to inflammation and pain, and the expression of genes and miRNAs involved in these pathways. In addition, the DED patients included herein were stratified based on the presence or absence of chronic ocular pain to investigate whether pain influences the long-term trajectory of disease-related biomarkers and clinical outcomes.

## 2. Results

**Figure 1:**
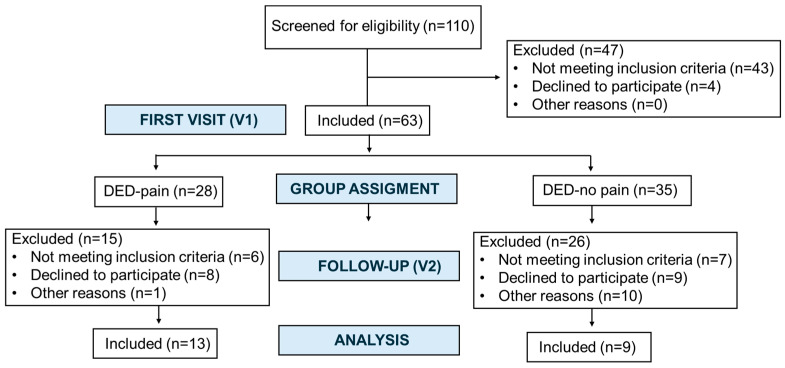
Flow of participants through the study from the first visit (V1) to the follow-up visit (V2).

This study corresponds to the second phase of a longitudinal study that initially included 63 patients at the first visit (V1) [9,20]. At the beginning of the second phase, all participants were invited to return for a follow-up visit (V2), which occurred after a mean interval of 3.82 ± 0.64 (range 2.83–3.92) years from V1. A total of 22 patients attended the V2. The flow of participants through the study is summarized in Figure 1. To address potential selection bias, we additionally compared baseline characteristics between participants who returned at V2 and those who did not (Supplementary Table S1). Of these, 19 (86.4%) were women and 3 (13.6%) were men. The overall mean age at V2 was 61.09 ± 7.95 (range, 43–71) years. Participants reported experiencing DED-related symptoms for a mean duration of 15.76 ± 12.52 (range, 5.33–53.58) years.

Patients were subsequently stratified into two groups based on the presence (DED-pain) or absence (DED-no pain) of chronic ocular pain. The DED-pain group included 13 patients, with a mean age of 61.38 ± 8.56 (range, 43–71) years, of whom 11 (84.6%) were women. The DED-no pain group included 9 patients, with a mean age of 60.67 ± 7.45 (range, 49–71) years, including 8 women (88.9%).

### 2.1. Clinical Evaluation

The treatments used to manage systemic conditions and DED at the time of V1 and V2 are summarized in Table 1. As this was an observational study, data on treatments used between visits were not collected.

Table 2 summarizes the results of symptom questionnaires assessing DED-related symptoms, ocular pain, anxiety, and depression in the entire patient cohort. A significant improvement in DED-related symptoms, as assessed by the Ocular Surface Disease Index (OSDI) questionnaire, was observed, whereas no other significant changes in symptom scores were detected between visits. Importantly, when considering the minimal clinically important difference for OSDI, 15 patients (68.2%) achieved a clinically meaningful improvement in symptoms.

Similarly, when stratified by group, patients in the DED-pain group showed a significant reduction in OSDI scores (Table 3). Although a decrease was also observed in the DED-no pain group, it did not reach statistical significance. No other statistically significant changes were observed between visits in either group.

Regarding ocular surface signs evaluated by slit-lamp biomicroscopy, no significant differences were found between visits in the overall cohort or within subgroups (Table 4).

Corneal sensitivity, assessed with the Belmonte noncontact esthesiometer, remained stable across visits in the entire sample, with no significant changes in mechanical, heat, or cold thresholds. Similarly, contact esthesiometry using the Cochet–Bonnet esthesiometer revealed no differences in sensitivity measurements before or after the application of topical anesthesia. Global Rating of Change scores were also consistent between visits (Table 5). Significant reductions in both total and small dendritic cell density were observed on in vivo confocal microscopy (IVCM), in the total sample analysis (Table 5).

However, when analyzed by group, no statistically significant differences were detected in any of the evaluated parameters (Table 6).

### 2.2. Sample Analysis

#### 2.2.1. Tear Cytokine and Substance P Analysis

Epidermal growth factor (EGF), IL-1Ra, IL-4, IL-8/CXCL8, monocyte chemoattractant protein (MCP)-1/CCL2, MCP-3/CCL7, growth-related oncogene (GRO), and substance P (SP) were detected in at least 90.9% of the subjects at both visits. Fractalkine/CX3CL1, IL-6, IL-10, MIP-1β/CCL4, and regulated on activation, normal T cell expressed and secreted (RANTES)/CCL5 exhibited detection rates ranging from 50 to 100% (Table 7). IL-1β, IL-2, IL-9, IL-17A, IFN-γ, MIP-1α/CCL3, nerve growth factor (NGF), and TNF-α were detected in less than 50% of samples in at least one visit and were therefore analyzed as qualitative variables (detected/undetected).

Quantitative analysis revealed significant decreases in the concentration levels of EGF, fractalkine/CX3CL1, and MCP1/CCL2 at V2 compared with V1, while SP levels significantly increased (Table 7). Among qualitatively analyzed molecules, IL-2, IL-9, and IL-17A showed significantly higher detection percentages at V2, whereas TNF-α and NGF showed significantly lower detection rates.

When patients were stratified into groups, the significant changes observed in the overall cohort were confirmed. In both the DED-pain and DED-no pain groups, EGF levels significantly decreased between visits. Specifically, EGF concentrations declined from 1730.69 ± 893.71 pg/mL at V1 to 935.46 ± 548.38 pg/mL at V2 in the DED-pain group (*p* < 0.001) and from 2187.22 ± 873.73 pg/mL to 715.56 ± 310.81 pg/mL in the DED-no pain group (*p* = 0.018). SP levels significantly increased in both groups: from 1289.75 ± 713.85 pg/mL to 3954.58 ± 3638.18 pg/mL in the DED-pain group (*p* = 0.006) and from 997.38 ± 576.82 pg/mL to 4304.51 ± 2477.08 pg/mL in the DED-no pain group (*p* < 0.001).

#### 2.2.2. Gene Expression Analysis

The expression levels of the 25 analyzed genes, grouped according to their biological function, are shown in Table 8. Among these, only one gene, *NTRK1*, showed a statistically significant downregulation at V2 compared with V1.

In addition, when gene expression was analyzed separately by group, a significant downregulation of *NTRK1* was observed in the DED-no pain group (V1: 9.74 [6.49–12.99] vs. V2: 17.63 [14.75–20.51]; *p* = 0.05). In contrast, no significant differences were detected in the DED-pain group (V1: 11.88 [9.42–14.33] vs. V2: 14.51 [13.36–15.66]; *p*= 0.625). No other significant changes in gene expression were detected between visits in either group.

#### 2.2.3. miRNA Expression Analysis

Among the 12 miRNAs evaluated, a significant downregulation of miR-665 was observed at V2 compared with V1 (Table 9). However, when miRNA expression was analyzed separately by groups, no significant differences were found between visits.

## 3. Discussion

The overlap between DED-related symptoms and chronic-ocular-pain-related symptoms, combined with the frequent lack of correlation between clinical signs and patient-reported symptoms, significantly complicates early diagnosis and appropriate management of these conditions [21]. This diagnostic challenge underscores the urgent need for reliable biomarkers to improve early identification and monitor the progression of these coexistent and interrelated conditions. Accordingly, this study aimed to evaluate the longitudinal progression of patients with DED both as a whole and stratified by the presence or absence of chronic ocular pain, to better elucidate the impact of persistent pain on disease evolution. To date, and to our knowledge, this is the first study to longitudinally investigate the clinical, molecular, and gene and miRNA expression profiles of these patients.

Our results demonstrated a significant improvement in DED-related symptoms, both in the overall cohort and specifically within the DED-pain group. Ocular surface parameters and corneal sensitivity remained stable between visits, regardless of whether the analysis was conducted on the full sample or stratified by group. At the level of the corneal sub-basal nerve plexus, a significant reduction in total and small dendritic cell density was observed in the whole cohort, although this finding was not maintained when groups were analyzed separately. Changes in EGF and SP concentrations were found both in the total sample and in the group-based analysis. Additionally, alterations in fractalkine/CX3CL1 and MCP-1/CCL2 levels were found in the full cohort. For molecules analyzed qualitatively as detected and nondetected, significant differences were observed in the percentage of detection of IL-2, IL-9, IL-17A, TNF-α and NGF. At the gene and miRNA expression level, *NTRK1* gene expression was downregulated in the overall cohort and in the DED-no pain group, while miR-665 expression was significantly downregulated only in the total sample.

Patients included in this study exhibited a severe degree of DED symptoms at V1. When analyzing the full cohort, a significant improvement was found in DED-related symptoms over time, as measured with the OSDI questionnaire, decreasing from severe to moderate intensity, with 68.2% of patients achieving the minimal clinically important difference for OSDI values. This improvement was also significant within the DED-pain group, with symptoms decreasing from a severe to a moderate level. Although the DED-no pain group did not show statistically significant change, symptom scores also decreased from severe to moderate. While this difference did not reach statistical significance—likely because of the smaller sample size—it may still reflect a clinically meaningful improvement. Patients with chronic ocular pain reported a moderate intensity of pain, and this intensity remained stable throughout the study. Approximately 4 years after their first visit, most patients reported similar symptoms to those experienced at V1, as assessed by the Change in Dry Eye Symptoms Questionnaire (CDES-Q). This outcome may be partly explained by the nature of the questionnaire, which assesses global symptom perception rather than distinguishing between specific complaints such as dryness or pain. In patients with chronic ocular pain, the lack of improvement in pain intensity may have influenced their overall perception of symptom stability, despite the OSDI score improvement found. These results are in line with previous studies showing that individuals with ocular pain tend to report more severe and persistent DED symptoms over time, which is consistent with our findings [22,23]. Furthermore, pain has been consistently identified as one of the most prevalent symptoms in chronic DED [23]. This is particularly relevant since patients exhibiting chronic ocular pain often respond inadequately to first-line topical therapies and may benefit from multimodal treatment strategies targeting the ocular sensory system [24]. In this context, the observed improvement in DED symptoms across the full cohort may reflect a positive response to the standard topical therapies these patients were using. However, the persistence and lack of improvement in ocular pain symptoms may be attributable to the absence of specific treatments targeting ocular pain mechanisms in the DED-pain group. While patients in our sample were treated with standard topical therapies, those with chronic ocular pain may require more advanced treatment options. No changes were observed in the topical or systemic medications that these subjects were receiving. It is important to acknowledge that this was an observational study, and patients were not monitored at our center between the two study visits. During that period, they continued their clinical care at their referring centers, where it can be assumed they received the best available treatment. Nonetheless, our findings highlight that chronic ocular pain, although closely related to DED, is often under-recognized as a specific component within this condition, leading to suboptimal management when not properly identified. This underscores the need to increase awareness of ocular pain mechanisms and promote individualized, multimodal treatment approaches.

No significant changes were found in slit-lamp-based ocular surface signs [24], indicating the stability of these clinical parameters over time. Consistently with our findings, a previous longitudinal study in patients with chronic ocular pain and DED after corneal refractive surgery reported only an improvement in corneal staining, which was not clinically significant, and no other relevant changes in ocular surface signs over time [25].

Similarly, corneal sensitivity showed no significant changes between visits, regardless of the measurement method used or the study group. Alterations in corneal sensitivity are common in DED patients [26,27,28]. However, the nature of these sensory changes remains controversial because of inconsistent findings in the literature. While some studies have reported corneal hypersensitivity [26], others have documented hyposensitivity when using noncontact esthesiometers to assess corneal sensitivity [27]. These discrepancies have been attributed to differences in measurement techniques, disease severity, and DED subtypes [28]. Despite this variability, there is a consensus that patients with DED exhibit a decreased corneal mechanical sensitivity when assessed using the Cochet–Bonnet esthesiometer [29,30,31]. Additionally, corneal hypersensitivity has been linked to increased DED-related symptoms and ocular pain [32].

Similarly, sub-basal corneal nerve parameters remained stable between visits, consistently with the absence of differences in corneal sensitivity. Interestingly, a reduction in total corneal dendritic cell density, primarily due to a decrease in the number of small dendritic cells, was observed across all patients. However, stratification by patient groups revealed no significant changes, suggesting that ocular pain does not significantly influence these cellular alterations, which appear to be primarily related to the underlying DED. Since increases in dendritic cell density are generally considered as markers of inflammation or immune activation, the observed reduction may reflect a decrease in immune activity at the ocular surface [28,33]. Previous studies have shown increased dendritic cell density in the central corneas of DED patients compared with healthy controls, particularly those with severe symptoms [34,35,36]. Although previous studies have associated higher microneuroma density with chronic ocular pain in patients with DED [22,37], no significant differences were observed between visits in our study. These findings underscore the potential utility of IVCM and the study of corneal dendritic cell analysis as valuable biomarkers for monitoring disease progression and inflammatory status in DED patients.

Tear film analysis of the entire cohort revealed significant decreases in the concentrations of EGF, fractalkine/CX3CL1, and MCP-1/CCL2, along with a significant increase in SP levels. Analysis by pain classification indicated that both DED-pain and DED-no pain patients showed reduced EGF and elevated SP concentration.

EGF levels were consistently reduced in the overall sample and in each of the two subgroups. EGF is a key growth factor involved in epithelial cell proliferation and differentiation, as well as wound healing, playing an essential role in maintaining ocular surface integrity [38]. Furthermore, EGF is a cytokine secreted by the lacrimal glands and has been shown to inversely correlate with Schirmer test results [39]. Previous studies have reported reduced EGF levels in tears of DED patients [39,40]. Comparison of our findings with the mean concentrations reported in healthy controls from a previous study conducted by our research group suggests that EGF concentration remained altered over time in this population. In healthy individuals, a mean decrease of approximately 6.3% in tear EGF concentration was observed over a similar follow-up period of time and mean age, likely reflecting physiological aging. In contrast, our cohort exhibited a markedly higher decline of approximately 56% between visits [41], indicating that the observed reduction was unlikely to be solely attributable to normal aging processes. Further research is needed to determine the clinical significance of this sustained decrease.

There was a significant reduction in fractalkine/CX3CL1 in the overall patient analysis. Fractalkine/CX3CL1 is a chemokine critical for neuroimmune communication, acting as a strong chemoattractant for immune cells. Persistent elevation of fractalkine/CX3CL1 has been linked to enhanced neuroinflammation and central sensitization mechanisms involved in chronic pain [42,43]. While our group previously reported increased fractalkine levels in DED patients compared with healthy controls [44], the present study found a decrease in fractalkine concentrations over time, potentially reflecting a reduction in neuroinflammatory activity with time in these patients.

Similarly, a decrease in MCP-1/CCL2 concentration was observed in the overall patient analysis. MCP-1/CCL2, a key chemokine involved in monocyte recruitment to sites of tissue injury or inflammation, is also implicated in pain modulation [45,46]. Prior studies demonstrated that spinal administration of TNF-α induces pain hypersensitivity and upregulates MCP-1, supporting its role in neuroinflammatory sensitization [47]. The decrease observed in this study may indicate a transition from an active neuroinflammatory state to a reduced inflammatory condition.

An increase with time in SP was found in both study groups and in the overall cohort. SP is a neuropeptide expressed by corneal nerves that regulates neuroinflammation and pain pathways [28,48,49]. Increased tear SP levels have been observed in DED patients, with concentrations correlating positively with symptom severity measures such as in OSDI score, photophobia frequency, and blurred vision [50]. However, another recent study found a negative correlation between SP and OSDI scores, which aligns with the reduction in OSDI scores and the increase in SP levels over time found in our study [51]. Also in line with our findings, experimental models of DED in mice have shown that SP expression in ocular surface tissues and trigeminal ganglia increases during the course of DED [52]. Interestingly, DED patients often report symptoms disproportionate to clinical signs [52], a discrepancy especially marked in those with chronic ocular pain, particularly in cases with neuropathic pain component [53]. Increased levels of SP have been shown to impair the function of regulatory T cells, which typically act to suppress effector immune cell activity, thereby sustaining chronic inflammation [49]. Previous research has pointed to SP as a promising target for therapeutic intervention in DED [54,55].

On the other hand, significant differences in the detection percentages of TNF-α, NGF, IL-2, IL-9, and IL-17A were found. The results of the present study showed a decrease in TNF-α detection over time. TNF-α is a well-established key mediator of inflammatory pain and plays a crucial role in chronic inflammatory diseases such as rheumatoid arthritis, where TNF-α inhibitors are widely used as effective treatments. Moreover, TNF-α is recognized as a proinflammatory and pronociceptive cytokine that is rapidly upregulated following tissue injury [56]. Its increased expression has also been reported in DED patients [38]. In our study, the reduction in TNF-α detection over time coincided with improvement in DE-related symptoms. Likewise, the NGF detection rate decreased in V2 compared with V1. NGF is a neurotrophic factor critically involved in the pathophysiology of inflammatory pain and has emerged as a promising therapeutic target for chronic pain management [57]. Additionally, NGF plays an important role in corneal nerve regeneration after corneal surgery [58,59]. In contrast, the detection rates of IL-2, IL-9, and IL-17A increased over time in our cohort, consistently with findings reported in other studies involving patients with DED [38,60]. However, since their percentage of detection was lower than 50%, no further conclusions could be obtained from their concentration.

To gain further insight into the molecular mechanisms underlying ocular surface inflammation and pain and their persistence over time, we examined both gene and miRNA expression profiles in this study. We found a downregulation of the *NTRK1* gene, which encodes for tyrosine kinase receptor (Trk)A, the high-affinity receptor for NGF. Neurotrophins and their corresponding TRKs, encoded by the *NTRK* gene family (*NTRK1*, *NTRK2*, *NTRK3*), are essential for the development and maintenance of both the central and peripheral nervous systems [61]. As the high-affinity NGF receptor, *NTRK1* plays a crucial role in mediating inflammatory and neuropathic pain by sensitizing sensory neurons [62]. Interestingly, when patients were analyzed based on the presence of chronic ocular pain, the downregulation of *NTRK1* was observed only in those without pain, while patients reporting chronic pain showed no significant change in *NTRK1* expression. This finding may suggest a compensatory regulatory mechanism whereby individuals without chronic ocular pain retain or increase TrkA-mediated signaling, possibly contributing to tissue repair or protective neuroimmune modulation. On the other hand, in patients with stablished chronic pain, the lack of TrkA downregulation might be involved in maintenance of pain; actually, several chronic pain states are associated with increased TrkA immunoreactivity and enhanced NGF/TrkA signaling [63,64,65]. Notably, pharmacological inhibition of the NGF–TrkA pathway is currently under investigation as a therapeutic strategy for various chronic pain syndromes [66], highlighting the clinical relevance of this signaling axis. Regarding miRNA expression analysis, we observed a downregulation of miR-665 across all patient samples. Its upregulation has been previously associated with neuroprotection and modulation of inflammatory responses [67,68], whereas its downregulation has been shown to reduce both pain sensitivity and inflammatory responses in neuropathic pain animal models [69]. To our knowledge, this is the first study reporting miR-665 expression changes in the context of DED, highlighting the novelty of this finding for ocular-surface research. Therefore, miR-665 could represent a promising therapeutic target for the treatment of chronic ocular pain.

This study has some limitations. Because of the study’s observational nature, treatments received and disease progression during the intervals between visits were not systematically recorded. As these patients require frequent follow-ups and often attend different centers across various cities, it was unfortunately not possible to access their medical records. Therefore, potential changes in medication that could have influenced the observed symptom improvement and molecular shifts cannot be analyzed. A control group would not be ethically feasible, which prevented monitoring the natural progression of the disease over time. Additionally, the relatively small sample size may limit the generalizability of our findings. Moreover, dividing the sample into subgroups further reduced the statistical power, which may explain the loss of significance observed in some parameters measured. In addition, we cannot completely exclude that the decrease in cytokine detection percentages observed at V2 was influenced by the lower sensitivity of the second assay kit. However, this would not affect cases in which detection percentages increased. Moreover, different customized assay kits were used for V1 and V2 (SPR 1549 and SPR 2141, respectively). Although both were based on the HCYTA-60 K Milliplex Kit, these differences could contribute to variability in detection values between visits. Finally, the stability over time observed in some parameters could reflect either effective long-term management of the ocular surface or that the follow-up period, although relatively long, was still insufficient to detect progressive changes.

Despite the relatively small sample size, the main strength of this study is its long-term longitudinal design, which enables a thorough evaluation of disease progression over time. This approach provides novel evidence of the clinical and molecular changes in patients with DED, with and without chronic ocular pain. By integrating clinical assessments, tear biomarkers, confocal microscopy, and gene and miRNA expression analysis, our findings contribute to a better understanding of the underlying neuroinflammatory mechanisms and highlight potential biomarkers for monitoring disease progression. These results reinforce the importance of differentiating ocular pain as a distinct clinical entity and may guide future research toward more personalized and mechanism-based therapeutic strategies.

## 4. Materials and Methods

This prospective observational study included patients with DED. Ethical approval was obtained from the East Valladolid Health Area Ethics Committee (Spain), and the study was conducted in accordance with the tenets of the Declaration of Helsinki. All participants provided written informed consent after receiving detailed information about the study objectives.

### 4.1. Participants and Study Design

Patients with DED attended two visits (V1 and V2) separated by a minimum interval of two years, following the same evaluation protocol. The initial visit was conducted as part of a previous study [9,20], and eligibility for V2 required prior enrollment in that study. The same exclusion criteria were applied at both visits and included: the presence of any ocular surface disease other that DED or chronic ocular pain within the previous 3 months; a history of ocular, periocular, or orbital surgeries; diagnosis of a systemic condition with potential ocular involvement within the previous 3 months; initiation of any systemic therapy that could affect ocular surface health in the last 3 months; initiation of lacrimal punctum occlusion within the previous 3 months; contact lens wear in the 7 days before to the study (for contact lens users); and the use of topical medications or lubricants within 24 and 12 h, respectively, before the study visit.

DED diagnosis was determined based on the presence of DED-related symptoms, defined as an OSDI questionnaire score > 13, along with at least two of the following signs present in both eyes: (1) fluorescein tear break-up time (TBUT) ≤ 7 s; (2) corneal fluorescein staining ≥ grade 1 (Oxford scale), (3) conjunctival lissamine green staining ≥ grade 1 (Oxford scale); or (4) Schirmer test with topical anesthesia ≤ 5 mm in 5 min [39,70,71].

Patients were subsequently classified into two groups: those with DED and chronic ocular pain (DED-pain) and those with DED without chronic ocular pain (DED-no pain). Ocular pain was defined as a score of ≥2 on both Numerical Rating Scale and Wong–Baker Faces Pain Rating Scale [72]. Pain was considered chronic when it persisted for 3 months or longer [73].

### 4.2. Clinical Evaluation

All participants underwent assessment after a 20 min period under normal controlled environmental conditions (23 °C, 50% relative humidity, and no localized air flow) in the Controlled Environment Laboratory (CELab) (www.visionrd.com/celab/, accessed on 5 September 2025) [74].

#### 4.2.1. Medical History and Symptom Assessment

A comprehensive medical history was recorded to evaluate both ocular and systemic health and to confirm eligibility based on the exclusion criteria. Participants then completed standardized questionnaires to assess the severity of DED-related symptoms, including the OSDI questionnaire, which categorizes symptom severity as asymptomatic (0–12), mild (13–22), moderate (23–32), or severe (33–100), and the modified Single-Item Dry Eye Questionnaire (mSIDEQ), which evaluates the frequency of symptoms related to DE (score range: 0–28) [75]. In addition to mean changes, the minimal clinically important difference for OSDI was calculated for each participant, defined as 4.5–7.3 points for patients with mild to moderate disease and 7.3–14.3 for those with severe intensity. The proportion of patients achieving an improvement greater than or equal to the thresholds was calculated [76]. The presence and intensity of ocular pain were assessed using the NRS, a scale from 0 to 10 in which scores of 0–1 indicate no pain; 2–4, mild pain; 5–7, moderate pain; and 8–10, severe pain. The Wong–Baker Faces Pain Rating Scale was also administered, using facial expressions to visually represent increasing levels of pain, from 0 (no pain) to 10 (unbearable pain) (https://wongbakerfaces.org/) [72,77,78].

Levels of anxiety and depression were measured with the Hospital Anxiety and Depression Scale, which includes two subscales (anxiety and depression), each scored from 0 to 21, with total scores ranging from 0 to 42. Scores of 0–7 are considered normal; 8–10, borderline; and 11–21, indicative of clinically significant symptoms [79,80].

Finally, changes in ocular pain and DE-related symptoms were evaluated using the CDES-Q, consisting of two parts: CDES-Q1, which assesses symptom progression (improved, worsened, or unchanged), and CDES-Q2, which rates the magnitude of change on a scale from 0 to 10 [81].

#### 4.2.2. Tear Sample Collection

Basal tear samples were collected from the external canthus using glass capillary micropipettes (Drummond Scientific Co., Broomall, PA, USA), taking care to avoid inducing reflex tearing. For cytokine quantification, a 1 µL basal tear sample was obtained from one randomly selected eye and diluted at a 1:10 ratio in a cryotube prefilled with 9 µL of chilled Milliplex Cytokine Assay Buffer (Merck Millipore, Burlington, MA, USA). In parallel, a 2 µL sample from the contralateral eye was collected for SP analysis and diluted 1:25 in the appropriate SP assay buffer (Cayman Chemical, Ann Arbor, MI, USA). Throughout the study visit, all tear samples were stored at 4 °C and promptly frozen at −80 °C until biochemical analysis was performed.

#### 4.2.3. Ocular Surface Evaluation

The ocular surface of both eyes was evaluated using a slit lamp (SL-D7, Topcon Corporation, Tokyo, Japan). Bulbar conjunctival hyperemia and blepharitis were evaluated with the Efron scale (range, 0–4) [82]. Lid-parallel conjunctival folds were assessed nasally and temporally and scored on a 0–3 scale [83]. Following the instillation of prewetted sodium fluorescein strips (I-DEW flo, Entod Research Cell UK Ltd., London, UK), TBUT was measured three consecutive times, and the mean value was used for analysis [21]. Two minutes after TBUT measurement, corneal fluorescein staining was graded according to the Oxford grading scale (range, 0–5) [84]. Conjunctival staining was subsequently evaluated using lissamine green strips (I-DEW green, Entod Research Cell UK Ltd., London, UK) and scored with the same scale [84]. Lid wiper epitheliopathy was evaluated using the method described by Korb et al., with scores ranging from 0 to 3, based on the average of horizontal and vertical involvement [85]. Meibomian gland function was assessed by evaluating both meibum quality and expressibility. Digital pressure was applied to the upper and lower eyelid margins. Secretion quality was graded on a 0–3 scale according to Bron et al. [86], and expressibility was evaluated using the 0–3 scale described by Shimazaki et al. [87].

Noncontact esthesiometry was performed using a noncontact Belmonte’s gas esthesiometer to determine mechanical and thermal sensitivity thresholds (heat and cold) following protocols previously described by our research group [88]. Additionally, mechanical sensitivity thresholds were assessed using the Cochet–Bonnet contact esthesiometer (Luneau Ophthalmology, Chartres, Paris, France), both before and after instillation of topical anesthetic (1 drop of 0.1% tetracaine and 0.4% oxibuprocaine; Anestésico Doble Colirio, Alcon Cusí, El Masnou, Spain), following standard procedures. Corneal sensitivity threshold was defined as the longest filament length (range, 0–60 mm) that elicited a positive response [70,89]. The anesthetic challenge test was conducted immediately after postanesthetic Cochet–Bonnet esthesiometry. The Global Rating Change (GRC) scale was used to assess changes in symptom intensity, ranging from −5 (complete recovery) to 0 (no change) to +5 (marked worsening) [89].

Basal tear production was assessed with the Schirmer’s test with topical anesthesia [70].

Following the instillation of a topical anesthetic, IVCM was conducted bilaterally using the Heidelberg Retina Tomograph III coupled with the Rostock Cornea Module (Heidelberg Engineering GmbH, Heidelberg, Germany). One eye was randomly selected for analysis, and three high-quality (same for V1 and V2), nonoverlapping images of the central sub-basal nerve plexus were selected and evaluated. Quantitative analysis was carried out using the ImageJ software, version 1.54g4 along with the NeuronJ plugin (https://imagescience.org/meijering/software/neuronj, accessed on 7 September 2025). The analysis included the following parameters: (1) total number of nerves (n/mm^2^), defined as the sum of visible nerve fibers per image; (2) nerve density (mm/mm^2^), measured as the cumulative length of all nerves within each frame; (3) average nerve length (mm/mm^2^) per image; (4) density of nerve branch points (n/mm^2^), referring to the count of nerve bifurcations per image; (5) nerve tortuosity, graded on a 0–4 scale according to the Oliveira-Soto and Efron classification [90]; (6) microneuroma density (n/mm^2^), assessed by counting irregularly shaped terminal nerve endings; (7); total dendritic cell density (n/mm^2^), corresponding to the number of hyperreflective dendriform structures identified—these were further classified into large (≥25 µm) dendritic cells, small (<25 µm) dendritic cells, and globular immune cells, the latter defined as round-shaped cells lacking dendrites [91,92]; and (8) image reflectivity, quantified as the mean optical density of the sub-basal plexus using the histogram tool in ImageJ. For each metric, the average from three selected images was used in the final analysis. All images from V2 were evaluated by a single trained observer, who was trained using the same protocol as for V1, and all analyses were performed following the same procedures. Excellent interobserver reliability (intraclass correlation coefficient > 0.90 for all nerve parameters except tortuosity, which showed moderate agreement with an intraclass correlation coefficient of 0.63) was previously demonstrated in a previous study in the same patient population [93].

#### 4.2.4. Conjunctival Impression Cytology

Conjunctival impression cytology was performed under topical anesthesia to collect conjunctival cells. A polyethersulfone filter (Supor 200, pore size: 0.20 μm, diameter: 13 mm; Gelman Laboratory, Ann Arbor, MI, USA) was halved and gently applied with light pressure to the upper temporal bulbar conjunctiva, one per eye. The filters were then carefully removed and transferred, one to 350 µL of RLT buffer (Qiagen, Hilden, Germany) containing 1% 2-mercaptoethanol for gene expression analysis and the other to 350 µL of Qiazol buffer for miRNA expression analysis. Samples were stored at −80 °C until further analysis [13].

### 4.3. Sample Analysis

#### 4.3.1. Tear Analysis

Cytokine concentrations in tear samples were measured using X-MAP technology with two customized multiplexed immunobead-based assays: SPR 1549 (for samples in V1) and SPR 2141 (for samples in V2). These assays utilized the Custom 20-plex Magnetic Human Cytokine Milliplex MAP panels (Millipore, Merck, MA, USA) and were analyzed on a MAGPIX^®^ system (Luminex Corporation, Austin, TX, USA) following the manufacturer’s low-volume protocol, which uses 10 µL of either sample or standard per assay, as previously described [44]. A total of 20 cytokines were simultaneously quantified, including EGF, fractalkine/CX3CL1, IL-1β, IL-1Ra, IL-2, IL-4, IL-6, IL-8/CXCL8, IL-9, IL-10, IL-17A, MCP-1/CCL2, MCP-3/CCL7, TNF-α, IFN-γ, GRO, MIP-1α/CCL3, MIP-1β/CCL4, NGF, and RANTES/CCL5. Cytokine concentrations were determined by converting fluorescence readings into cytokine levels (pg/mL) using standard curves. The minimum detectable concentrations, according to the manufacturer’s guidelines, were 3.2 pg/mL for EGF, Fractalkine/CX3CL1, IL-1Ra, IL-2, IL-4, IL-8/CXCL8, IL-9, IL-10, IL-17A, MCP-3/CCL7, IFN-γ, GRO, MIP-1α/CCL3, MIP-1β/CCL4, NGF, and RANTES/CCL5; 6.14 pg/mL for IL-6; 6.4 pg/mL for TNF-α; and 9.6 pg/mL for IL-1β and MCP-1/CCL2.

#### 4.3.2. Gene Expression Analysis

The expression of 25 human genes involved in neuropathic and inflammatory pain was analyzed and is listed in Table 10 in alphabetical order. First, RNA was extracted from CIC samples through a commercial kit (RNeasy Micro Kit, Qiagen, Redwood City, CA, USA) and 20 ng of cDNA from each sample was synthesized (iScript cDNA Synthesis kit, BioRad Laboratories Inc., Hercules, CA, USA). Second, gene expression was calculated by real time (RT)-PCR in a 7500 thermocycler (Applied Biosystems, Waltham, MA, USA) using a customized PCR array (BioRad, Hercules, CA, USA), following the manufacturer’s instructions. Finally, relative gene expression data were calculated by the Δ cycle threshold (Ct) method using glyceraldehyde-3-phosphate dehydrogenase (*GAPDH*) as housekeeping reference gene [94]. Gene fold change between visits was calculated using the 2^(−ΔΔCt)^ method [95]. Values greater than 1 were considered upregulated, while values lower than 1 were considered downregulated. Fold regulation was calculated to express fold change values in a biologically meaningful way: when fold-change values were greater than one (upregulation), fold regulation was equal to the fold change; when fold-change values were less than one (downregulation), fold regulation was reported as the negative inverse of the fold change.

#### 4.3.3. MicroRNA Expression Analysis

The expression of 12 human miRNAs (20a-5p; 23b-3p; 29a-3p; 92b-3p; 99a-5p; 137-3p; 143-3p; 208a-3p; 302d-3p; 379-3p; 543; 665) related to pain and inflammation was analyzed. First, miRNA was extracted from CIC samples using a commercial kit (miRNeasy Mini Kit, Qiagen, Hilden, Germany), and 10 ng of cDNA from each sample was synthesized using a commercial kit (miRCURY^®^ LNA^®^ RT Kit, Qiagen). Second, miRNA expression was calculated through real time (RT)-PCR in a 7500 thermocycler (Applied Biosystem) using customized miRNA PCR Panels (Qiagen), following the manufacturer’s instructions. Finally, relative miRNA expression data were calculated by the ΔCt method, using hsa-miR-103a-3p as housekeeping reference miRNA, and miRNA fold change between visits was calculated using the 2^(−ΔΔCt)^ method as explained above.

### 4.4. Statistical Analysis

Statistical analysis was conducted using SPSS software version 26.0 (SPSS Inc., Chicago, IL, USA). Data from both visits were compared. For each patient, one eye was randomly selected and used consistently for the analysis. Quantitative variables were expressed as mean ± standard deviation; categorical variables, as percentages; and ordinal variables, as median with interquartile range (IQR).

The Shapiro–Wilk test was employed to examine the assumption of normality. When this assumption was met, comparisons between visits in the entire cohort were performed using parametric tests. In contrast, because of the limited number of subjects in each subgroup, nonparametric tests were used to assess within-group changes between visits in the DED-pain and DED-no pain groups.

Specifically, for quantitative variables with a normal distribution, the paired Student’s *t*-test was used, after checking homogeneity of variance with Levene’s test. For non-normally distributed, ordinal, or qualitative data, the Wilcoxon signed-rank test was applied.

In the analysis of tear molecules, only those with a detection rate ≥ 50% were considered for quantitative analysis. Undetected values were imputed using the minimum concentration value of the V1 standard curve, and concentrations were log-transformed (log 2) to normalize the distribution. Molecules detected at rates lower than 50% were not imputed and were analyzed as binary variables (detected vs. undetected) using the McNemar test.

For gene and miRNA expression analysis, nondetected values (Ct > 40) were assigned a value of 40. Gene and miRNA changes between visits were evaluated individually in each patient through the paired Student’s *t*-test.

The false discovery rate was controlled using the Benjamini–Hochberg correction method for multiple testing. *p*-values ≤ 0.05 were considered statistically significant.

## 5. Conclusions

This observational study captured the real-life evolution of patients with DED disease, with and without chronic ocular pain, within the context of everyday clinical care. No significant changes were observed in ocular surface clinical signs that could reliably monitor disease progression. However, IVCM revealed a reduction in the density of dendritic cells within the sub-basal nerve plexus, suggesting decreased immune activity or inflammation. Additionally, tear film analysis revealed significant molecular changes, including a decrease in levels of EGF, fractalkine/CX3CL1, and MCP-1/CCL2, alongside an increase in SP concentrations over time. The persistent increase in SP further highlights ongoing neuroimmune dysregulation and pain sensitization. Downregulation of *NTRK1* gene expression, particularly in the DED-no pain group may reflect a protective or compensatory neuroimmune mechanism in less severe stages of the disease. Moreover, the observed downregulation of miR-665 reinforces the presence of molecular adaptations associated with the chronicity of DED.

In conclusion, our findings indicate that although overall DED symptoms may improve with standard therapies, patients experiencing chronic ocular pain show persistent pain severity, underscoring the necessity to consider DED and ocular pain as distinct entities that require individualized, multimodal treatment approaches. These considerations are increasingly recognized in the literature [96].

## Figures and Tables

**Table 1:** Systemic and ocular topical treatments for dry eye disease at V1 and V2.

**Systemic Treatments**	**All (n = 22)**			**DED-Pain (n = 13)**			**DED-No Pain (n = 9)**		
	**V1**	**V2**	***p*-Value**	**V1**	**V2**	***p*-Value**	**V1**	**V2**	***p*-Value**
**Analgesics and NSAIDs**	8 (36.4)	3 (13.6)	0.125	6 (46.2)	3 (23.1)	0.375	2 (22.2)	0 (0)	0.500
**Antacids**	6 (27.3)	5 (22.7)	1.000	4 (30.8)	3 (23.1)	1.000	2 (22.2)	2 (22.2)	1.000
**Anticoagulants**	2 (9.1)	2 (9.1)	1.000	1 (7.7)	1 (7.7)	1.000	1 (11.1)	1 (11.1)	1.000
**Antihypertensives**	4 (18.2)	5 (22.7)	1.000	3 (23.1)	3 (23.1)	1.000	1 (11.1)	2 (22.2)	1.000
**Asthma treatments**	3 (13.6)	3 (13.6)	1.000	3 (23.1)	3 (23.1)	1.000	0 (0)	0 (0)	1.000
**Cholesterol-lowering drugs**	6 (27.3)	7 (31.8)	1.000	3 (23.1)	4 (30.8)	1.000	3 (33.3)	3 (33.3)	1.000
**Neuropsychiatric treatments**	3 (13.6)	2 (9.1)	1.000	2 (15.4)	1 (7.7)	1.000	1 (11.1)	1 (11.1)	1.000
Neurological disease treatments	0	1 (4.5)	1.000	2 (15.4)	1 (7.7)	1.000	0 (0)	0 (0)	1.000
Anxiolytics	2 (9.1)	1 (4.5)	1.000	1 (7.7)	0 (0)	1.000	0 (0)	0 (0)	1.000
Antidepressants	2 (9.1)	1 (4.5)	1.000	0 (0)	1 (7.7)	1.000	1 (11.1)	1 (11.1)	1.000
Antiepileptics	1 (4.5)	1 (4.5)	1.000	0 (0)	1 (7.7)	1.000	1 (11.1)	0 (0)	1.000
Other	1 (4.5)	2 (9.1)	1.000	1 (7.7)	0 (0)	1.000	1 (11.1)	1 (11.1)	1.000
**Immunosuppressants and corticosteroids**	3 (13.6)	0	0.250	2 (15.4)	3 (23.1)	1.000	2 (22.2)	0 (0)	0.500
**Hormonal and genitourinary treatments**	5 (22.7)	6 (27.3)	1.000	1 (7.7)	0 (0)	1.000	3 (33.3)	3 (33.3)	1.000
**Antidiabetic agents**	1 (4.5)	0	1.000	3 (23.1)	4 (30.8)	1.000	0 (0)	0 (0)	1.000
**Miscellanea**	5 (22.7)	6 (27.3)	1.000	3 (23.1)	4 (30.8)	1.000	2 (22.2)	2 (22.2)	1.000
Vitamins and food supplements	4 (18.2)	6 (27.3)	0.500	0 (0)	0 (0)	1.000	1 (11.1)	1 (11.1)	1.000
Muscle relaxants	1 (4.5)	0	1.000	0 (0)	1 (7.7)	1.000	1 (11.1)	1 (11.1)	1.000
Cardiovascular treatments	0	1 (4.5)	1.000	0 (0)	0 (0)	1.000	0 (0)	0 (0)	1.000
**Ocular topical treatments**	**All (n = 22)**			**DED-Pain (n = 13)**			**DED-No Pain (n = 9)**		
	**V1**	**V2**	***p*-Value**	**V1**	**V2**	***p*-Value**	**V1**	**V2**	***p*-Value**
**Lubricants**	15 (68.2)	14 (63.3)	1.000	9 (69.2)	10 (76.9)	1.000	6 (66.7)	4 (44.4)	0.500
Artificial tears	15 (68.2)	14 (63.3)	1.000	9 (69.2)	10 (76.9)	1.000	6 (66.7)	4 (44.4)	0.500
Gels	1 (4.5)	0 (0)	1.000	1 (7.7)	0 (0)	1.000	0 (0)	0 (0)	1.000
Ointments	1 (4.5)	1 (4.5)	1.000	1 (7.7)	1 (7.7)	1.000	0 (0)	0 (0)	1.000
**Eyelid hygiene**	8 (36.4)	4 (18.2)	0.125	5 (38.5)	2 (15.4)	0.250	3 (33.3)	2 (22.2)	1.000
**Punctal plugs**	3 (13.6)	2 (9.1)	1.000	2 (15.4)	2 (15.4)	1.000	1 (11.1)	0 (0)	1.000
**Blood derivates**	3 (13.6)	2 (9.1)	1.000	3 (23.1)	2 (15.4)	1.000	0 (0)	0 (0)	1.000
**Corticosteroids**	1 (4.5)	2 (9.1)	1.000	1 (7.7)	1 (7.7)	1.000	0 (0)	1 (11.1)	1.000
**Cyclosporine**	0 (0)	1 (4.5)	1.000	0 (0)	1 (7.7)	1.000	0 (0)	0 (0)	1.000

Data are presented as frequencies n (%). V1: visit 1; V2: visit 2; NSAIDs: nonsteroidal anti-inflammatory drugs.

**Table 2:** Changes in symptoms between V1 and V2 in the overall cohort (n = 22).

	V1	V2	*p*-Value
**OSDI** (0–100)	38.88 ± 19.31	25.09 ± 17.35	**0.002**
**mSIDEQ** (0–28)	13.68 ± 4.59	13.64 ± 4.69	1.000
**Intensity of pain** (NRS scale, 0–10)	3.98 ± 3.58	2.89 ± 3.48	0.378
**Intensity of pain** (WFPRS scale, 0–10)	4.00 ± 3.75	3.11 ± 3.53	0.445
**HADS questionnaire** (0–42)	8.55 ± 6.04	8.36 ± 4.45	1.000
Anxiety subscale (0–21)	5.82 ± 3.50	5.86 ± 2.96	1.000
Depression subscale (0–21)	2.73 ± 2.83	2.50 ± 2.15	1.000
**CDES-Q1**			
Better n (%)	-	5 (22.7)	-
Worse n (%)	-	6 (27.3)	-
Same n (%)	-	11 (50.0)	-
**CDES-Q2**			
Improvement intensity (0–10)	-	7.00 ± 2.32	-
Worsening intensity (0–10)	-	5.33 ± 0.82	-

Data are presented as mean ± standard deviation. V1: visit 1; V2: visit 2. CDES-Q: Change in Dry Eye Symptoms Questionnaire; OSDI: Ocular Surface Disease Index; mSIDEQ: Modified Single Item Dry Eye Questionnaire; NRS: Numerical Rating Scale; WFPRS: Wong–Baker Faces Pain Rating Scale; HADS: Hospital Anxiety and Depression Scale. *p*-values in bold indicate statistically significant changes from V1 to V2.

**Table 3:** Changes in symptoms between V1 and V2 in the subgroups.

	DED-Pain (n = 13)			DED-No Pain (n = 9)		
	V1	V2	*p*-Value	V1	V2	*p*-Value
**OSDI** (0–100)	44.42 ± 21.24	29.37 ± 20.24	**0.021**	30.87 ± 13.42	18.91 ± 10.12	0.336
**mSIDEQ** (0–28)	16.15 ± 3.91	15.00 ± 4.78	0.442	10.11 ± 2.85	11.67 ± 4.00	0.336
**Intensity of pain** (NRS scale, 0–10)	6.65 ± 1.84	4.50 ± 3.53	0.189	0.11 ± 0.33	0.56 ± 1.67	0.665
**Intensity of pain** (WFPRS scale, 0–10)	6.77 ± 2.09	4.96 ± 3.39	0.205	0.00 ± 0.00	0.44 ± 1.33	0.370
**HADS questionnaire** (0–42)	8.31 ± 4.84	10.00 ± 4.16	0.254	8.89 ± 7.77	6.00 ± 3.91	0.336
Anxiety subscale (0–21)	5.69 ± 3.04	6.85 ± 2.91	0.254	6.00 ± 4.27	4.44 ± 2.55	0.336
Depression subscale (0–21)	2.62 ± 2.33	3.15 ± 2.23	0.442	2.89 ± 3.59	1.56 ± 1.74	0.336
**CDES-Q1**						
Better n (%)	-	4 (30.8)	-	-	1 (11.1)	-
Worse n (%)	-	4 (30.8)	-	-	2 (22.2)	-
Same n (%)	-	5 (38.5)	-	-	6 (66.7)	-
**CDES-Q2**						
Improvement intensity (0–10)	-	7.75 ± 1.85	-	-	4.00	-
Worsening intensity (0–10)	-	5.50 ± 0.58	-	-	5.00 ± 1.41	-

Data are presented as mean ± standard deviation. DED: dry eye disease; V1: visit 1; V2: visit 2. CDES-Q: Change in Dry Eye Symptoms Questionnaire; OSDI: Ocular Surface Disease Index; mSIDEQ: Modified Single Item Dry Eye Questionnaire; NRS: Numerical Rating Scale; WFPRS: Wong–Baker Faces Pain Rating Scale; HADS: Hospital Anxiety and Depression Scale. *p*-values in bold indicate statistically significant changes from V1 to V2.

**Table 4:** Changes in ocular surface signs between V1 and V2 in the overall cohort and in the subgroups.

	All (n = 22)			DED-Pain (n = 13)			DED-No Pain (n = 9)		
	V1	V2	*p*-Value	V1	V2	*p*-Value	V1	V2	*p*-Value
Conjunctival hyperemia (Efron scale, 0–4)	1.00 [2.00–1.00]	2.00 [2.00–1.00]	0.460	1.00 [2.00–1.00]	2.00 [2.00–1.00]	0.579	1.00 [1.00–1.00]	2.00 [2.00–0.50]	0.988
Blepharitis (Efron scale, 0–4)	2.00 [2.25–1.00]	1.00 [2.00–1.00]	0.176	2.00 [2.50–1.00]	1.00 [2.00–1.00]	0.447	2.00 [2.50–1.00]	1.00 [2.00–0.50]	0.405
Nasal LIPCOF (0–3 scales)	2.00 [2.00–1.00]	1.00 [2.00–1.00]	0.127	2.00 [2.50–1.00]	1.00 [2.00–1.00]	0.360	2.00 [2.00–1.00]	1.00 [1.00–0.50]	0.420
Temporal LIPCOF (0–3 scales)	2.00 [2.00–1.00]	1.00 [2.00–1.00]	0.298	2.00 [2.00–1.50]	2.00 [2.00–1.00]	0.360	2.00 [2.00–0.50]	1.00 [2.50–0.50]	0.988
TBUT (seconds)	3.42 ± 1.33	3.50 ± 1.46	0.830	3.64 ± 1.44	3.13 ± 1.24	0.360	3.11 ± 1.16	4.04 ± 1.66	0.639
Corneal staining (Oxford scale, 0–5)	1.27 ± 0.94	0.78 ± 1.02	0.176	1.38 ± 0.96	1.00 ± 1.15	0.360	1.11 ± 0.93	0.44 ± 0.73	0.420
Conjunctival staining (Oxford scale, 0–5)	0.93 ± 0.71	0.59 ± 0.78	0.275	0.92 ± 0.70	0.23 ± 0.39	0.210	0.94 ± 0.77	1.11 ± 0.93	0.988
LWE (0–3 scale)	0.55 ± 0.63	0.82 ± 0.88	0.338	0.58 ± 0.64	1.08 ± 0.93	0.360	0.50 ± 0.66	0.44 ± 0.68	0.988
Meibum quality (0–3 scale)	1.00 [2.00–1.00]	2.00 [2.00–1.00]	0.275	1.00 [2.00–1.00]	2.00 [2.00–1.00]	0.360	2.00 [2.00–1.00]	2.00 [2.00–1.00]	0.988
Meibum Expressibility (0–3 scale)	2.00 [2.00–1.00]	2.00 [2.00–1.75]	0.176	2.00 [2.00–1.00]	2.00 [2.50–1.50]	0.290	2.00 [2.00–0.50]	2.00 [2.00–1.00]	0.988
Schirmer’s Test (mm)	10.41 ± 7.56	10.45 ± 9.94	0.350	11.08 ± 7.35	9.92 ± 9.79	0.360	9.44 ± 8.20	11.22 ± 10.69	0.999

Data are presented as mean ± standard deviation or as median [interquartile range]. DED: dry eye disease; V1: visit 1; V2: visit 2; LIPCOF: lid-parallel conjunctival folds: TBUT: tear break-up time; LWE: lid wiper epitheliopathy.

**Table 5:** Changes in corneal sensitivity and sub-basal nerve plexus between V1 and V2 in the overall cohort (n = 22).

	V1	V2	*p*-Value
**Noncontact corneal esthesiometry**			
Mechanical threshold (mL/min)	87.28 ± 41.96	114.76 ± 43.77	0.060
Heat threshold (°C)	2.12 ± 1.24	1.16 ± 0.73	0.060
Cold threshold (°C)	−2.23 ± 1.13	−1.31 ± 0.89	0.060
**Contact corneal esthesiometry**			
Before topical anesthesia (mm)	56.14 ± 7.86	55.68 ± 7.12	0.812
After topical anesthesia (mm)	8.81 ± 16.95	6.90 ± 12.09	0.812
**Anesthetic challenge test** (GRC scale; −5 to 5)	−1.77 ± 2.35	−1.16 ± 2.22	0.543
**Number of nerves** (n/mm^2^)	46.78 ± 20.87	51.89 ± 23.00	0.792
**Nerve density** (mm/mm^2^)	10,494.99 ± 4564.65	11,610.99 ± 4396.22	0.792
**Nerve length** (mm/mm^2^)	1414.71 ± 335.98	1518.27 ± 266.52	0.685
**Density of nerve branch points** (n/mm^2^)	26.89 ± 26.35	30.87 ± 21.26	0.827
**Nerve tortuosity** (0–4)	2.70 ± 0.71	2.65 ± 0.73	0.962
**Density of microneuromas** (n/mm^2^)	1.52 ± 2.40	1.99 ± 2.36	0.827
**Density of dendritic cells** (n/mm^2^)	74.05 ± 88.23	25.66 ± 23.79	**0.044**
Small dendritic cells (n/mm^2^)	56.82 ± 73.16	16.19 ± 20.17	**0.044**
Large dendritic cells (n/mm^2^)	15.81 ± 21.52	8.24 ± 9.65	0.169
Globular cells (n/mm^2^)	1.42 ± 2.97	1.23 ± 2.85	0.962
**Reflectivity**	102.67 ± 11.55	103.44 ± 12.55	0.962

Data are presented as mean ± standard deviation. V1: visit 1; V2: visit 2; GRC: Global Rating of Change. *p*-values in bold indicate statistically significant changes from V1 to V2.

**Table 6:** Changes in symptoms, ocular surface signs, corneal sensitivity, and sub-basal nerve plexus between V1 and V2 in the subgroups.

	DED-Pain (n = 13)			DED-No Pain (n = 9)		
	V1	V2	*p*-Value	V1	V2	*p*-Value
**Noncontact corneal esthesiometry**						
Mechanical threshold (mL/min)	91.57 ± 53.68	104.58 ± 38.82	0.915	81.56 ± 19.36	128.33 ± 48.54	0.090
Heat threshold (°C)	2.37 ± 1.20	1.40 ± 0.61	0.246	1.81 ± 1.28	0.87 ± 0.80	0.240
Cold threshold (°C)	−2.07 ± 1.15	−1.29 ± 0.77	0.342	−2.44 ± 1.15	−1.33 ± 1.09	0.240
**Contact corneal esthesiometry**						
Before topical anesthesia (mm)	55.77 ± 8.62	55.77 ± 7.03	0.886	56.67 ± 7.07	55.56 ± 7.68	1.000
After topical anesthesia (mm)	11.67 ± 20.71	9.17 ± 15.50	0.794	5.00 ± 10.00	3.89 ± 4.17	1.000
**Anesthetic challenge test** (GRC scale; −5 to 5)	−2.31 ± 2.18	−1.50 ± 2.35	0.760	−1.00 ± 2.50	−0.67 ± 2.06	1.000
**Number of nerves** (n/mm^2^)	41.99 ± 17.02	53.04 ± 25.64	0.209	53.70 ± 24.85	50.23 ± 19.94	0.844
**Nerve density** (mm/mm^2^)	9508.85 ± 4523.93	11,734.28 ± 4538.24	0.209	11,919.40 ± 4486.96	11,432.90 ± 4447.34	0.825
**Nerve length** (mm/mm^2^)	1387.18 ± 381.08	1515.77 ± 330.36	0.828	1454.48 ± 274.76	1521.88 ± 150.79	0.825
**Density of nerve branch points** (n/mm^2^)	19.79 ± 16.63	33.33 ± 24.03	0.209	37.15 ± 34.75	27.31 ± 17.23	0.825
**Nerve tortuosity** (0–4)	2.60 ± 0.75	2.77 ± 0.67	0.759	2.83 ± 0.65	2.48 ± 0.82	0.825
**Density of microneuromas** (n/mm^2^)	2.08 ± 2.79	1.76 ± 1.87	0.828	0.69 ± 1.47	2.31 ± 3.03	0.825
**Density of dendritic cells** (n/mm^2^)	82.53 ± 110.21	20.03 ± 26.47	0.209	61.81 ± 43.91	33.80 ± 17.58	0.600
Small dendritic cells (n/mm^2^)	64.10 ± 89.76	12.98 ± 23.94	0.209	46.30 ± 41.86	20.83 ± 12.93	0.825
Large dendritic cells (n/mm^2^)	16.83 ± 25.80	6.57 ± 8.06	0.209	14.35 ± 14.60	10.65 ± 11.67	0.825
Globular cells (n/mm^2^)	1.60 ± 2.71	0.48 ± 1.25	0.262	1.16 ± 3.47	2.31 ± 4.09	0.825
**Reflectivity**	99.76 ± 9.01	100.69 ± 14.14	0.759	106.87 ± 13.95	107.41 ± 9.16	0.844

Data are presented as mean ± standard deviation. DED: Dry Eye Disease; V1: visit 1; V2: visit 2; GRC: Global Rating of Change.

**Table 7:** Tear detection rates and concentrations of the 20 analyzed cytokines and substance P at V1 and V2 in the total study population.

		V1	V2		
	Detection Rate (%)	Concentration (pg/mL)	Detection Rate (%)	Concentration (pg/mL)	*p*-Value
EGF	100	1917.45 ± 894.45 (1520.88–2314.03)	100	845.50 ± 469.99 (637.12–1053.88)	**<0.001 ^a^**
Fractalkine/CX3CL1	50	1359.33 ± 1871.83 (529.40–2189.25)	100	1061.55 ± 583.81 (802.70–1320.39)	**0.020 ^a^**
IL-1β	*45.5*	*-*	*31.8*	*-*	0.664 ^b^
IL-1Ra	100	6846.06 ± 11,331.55 (1821.93–11,870.19)	100	7060.59 ± 8099.22 (3469.60–10,651.58)	0.299 ^a^
IL-2	*4.5*	-	63.6	30.02 ± 23.11 (19.77–40.27)	**0.004 ^b^**
IL-4	90.9	236.72 ± 328.95 (90.87–382.57)	95.5	405.65 ± 328.95 (155.43–655.87)	0.212 ^a^
IL-6	63.6	58.38 ± 88.34 (19.17–97.51)	59.1	90.00 ± 128.38 (33.08–146.92)	0.516 ^a^
IL-8/CXCL8	100	975.47 ± 3001.24 (−355.20–2306.15)	100	349.35 ± 509.40 (123.50–575.21)	0.971 ^a^
IL-9	*9.1*	-	59.1	30.02 ± 23.11 (19.77–40.27)	**0.004 ^b^**
IL-10	59.1	21.80 ± 26.85 (9.90–33.71)	68.2	87.53 ± 70.83 (56.13–118.94)	0.084 ^a^
IL-17A	*0*	-	68.2	45.92 ± 32.01 (31.73–60.12)	**<0.001 ^b^**
MCP-1/CCL2	100	801.95 ± 733.80 (476.61–1127.30)	95.5	492.19 ± 363.47 (331.03–653.34)	**0.020 ^a^**
MCP-3/CCL7	95.5	251.88 ± 138.45 (190.49–313.26)	100	272.22 ± 284.51 (146.07–398.36)	0.930 ^a^
TNF-α	59.1	9.25 ± 16.19 (2.07–16.42)	*18.2*	*-*	**0.024 ^b^**
IFN-γ	*27.3*	-	59.1	29.54 ± 22.37 (19.62–39.46)	0.087 ^b^
GRO	100	6592.32 ± 9261.33 (2486.07–10,698.56)	100	5223.36 ± 4675.23 (3150.48–7296,25)	0.690 ^a^
MIP-1α/CCL3	*9.1*	*-*	*4.5*	*-*	1.000 ^b^
MIP-1β/CCL4	59.1	71.20 ± 268.24 (−47.73–190.13)	59.1	14.26 ± 11.68 (9.08–19.44)	1.000 ^a^
NGF	77.3	8.09 ± 8.81 (4.18–11.99)	*36.4*	*-*	**0.035 ^b^**
RANTES/CCL5	86.4	82.68 ± 82.67 (46.03–119.34)	68.2	124.84 ± 89.03 (85.36–164.31)	1.000 ^a^
SP	100	1172.80 ± 662.72 (862.64–1482.96)	100	4094.55 ± 3155.11 (2617.92–5571.19)	**<0.001 ^a^**

Detection rates are expressed as percentages, and concentrations are presented as means (95% confidence interval) pg/mL. Molecules with a percentage of detection lower than 50% are in italics. ^a^ *p*-values correspond to comparisons of concentrations levels between both visits. ^b^ *p*-values correspond to comparison of detection rates between both visits. V1: visit 1; V2: visit 2; EGF: epidermal growth factor; IL: interleukin; IL-1Ra: interleukin-1 receptor antagonist; MCP: monocyte chemoattractant protein; TNF: tumor necrosis factor; IFN: interferon; GRO: growth related oncogene; MIP: macrophage inflammatory protein; NGF: nerve growth factor; RANTES: regulated on activation normal T cell expressed and secreted; SP: substance P. *p*-values in bold indicate statistically significant changes from V1 to V2.

**Table 8:** Changes in gene expression between V1 and V2.

Category		Gene	ΔCt V1	ΔCt V2	Fold Change	Fold Regulation	*p*-Value
Modulation of pain responses	Inflammation	*BDKRB1*	11.21 (8.95–13.49)	15.38 (13.09–17.68)	0.06	−18.00	0.125
		*IL1A*	18.21 (16.38–20.03)	18.13 (16.55–19.71)	1.06	1.06	1.000
		*IL2*	12.48 (10.74–14.23)	12.88 (12.20–13.56)	0.76	−1.32	1.000
		*CALCA*	11.62 (9.09–14.15)	14.09 (12.35–15.83)	0.18	−5.54	0.396
		*IL6*	10.79 (9.24–12.33)	11.39 (9.84–12.95)	0.66	−1.52	0.833
		*CCL2*	15.96 (13.86–18.05)	16.57 (14.74–18.39)	0.66	−1.52	1.000
		*ITGAM*	14.74 (12.50–16.98)	13.24 (11.51–14.97)	2.83	2.83	0.605
		*TAC1*	19.36 (17.82–20.90)	19.17 (17.98–20.35)	1.14	1.14	1.000
		*CD200*	12.58 (11.45–13.71)	14.76 (12.72–16.80)	0.22	−4.53	0.295
		*TACR1*	18.89 (16.94–20.84)	19.49 (18.84–20.13)	0.66	−1.52	1.000
		*TNF*	12.03 (9.84–14.21)	12.14 (10.23–14.05)	0.93	−1.08	1.000
		*CX3CR1*	10.82 (8.18–13.46)	10.95 (9.85–12.04)	0.91	−1.09	1.000
		*IL18*	4.68 (3.92–5.44)	4.58 (4.28–4.88)	1.07	1.07	1.000
	Neurotrophins	*BDNF*	7.86 (7.02–8.69)	8.94 (7.55–10.33)	0.47	−2.11	0.295
		*NGF*	14.79 (12.78–16.81)	15.39 (14.24–16.54)	0.66	−1.52	1.000
		*NTRK1*	11.21 (9.38–13.04)	15.48 (14.22–16.75)	0.05	−19.29	**0.025**
	Inflammation and neurotransmitters	*PENK1*	12.36 (9.57–15.14)	15.41 (13.05–17.76)	0.12	−8.28	0.295
Conduction of pain	Opioid receptors	*OPRM1*	19.49 (17.93–21.05)	19.85 (19.22–20.48)	0.78	−1.28	1.000
		*OPRD1*	19.56 (18.00–21.12)	19.85 (19.22–20.48)	0.82	−1.22	1.000
		*OPRK1*	13.73 (12.00–15.45)	14.25 (13.44–15.06)	0.70	−1.43	1.000
	Ion channels	*TRPV3*	13.74 (12.25–15.23)	16.43 (15.08–17.78)	0.15	−6.45	0.125
		*TRPA1*	17.90 (16.14–19.66)	19.26 (18.29–20.24)	0.39	−2.57	0.605
		*TRPV1*	9.28 (8.26–10.30)	8.63 (7.34–9.91)	1.57	1.57	0.675
	Cannabinoid receptors	*CNR2*	12.40 (9.65–15.14)	17.22 (15.15–19.29)	0.04	−28.25	0.125
Synaptic transmission	Eicosanoid metabolism	*PTGS1*	14.37 (12.56–16.19)	14.84 (12.90–16.79)	0.72	−1.39	1.000

Data are expressed as mean ΔCt (cycle threshold (Ct) gene of interest—Ct *GAPDH*) values (95% CI) for each gene. Gene fold change between visits was calculated using the 2^(−ΔΔCt)^ method; when fold-change values were greater than one (upregulation), fold regulation was equal to the fold change; when fold-change values were less than one (downregulation), fold regulation is reported as the negative inverse of the fold change. Note that high values of ΔCt indicate lower gene expression, whereas low ΔCt values indicate higher gene expression. Significant *p*-values are denoted in bold.

**Table 9:** Changes in miRNA expression between V1 and V2.

miRNA	ΔCt V1	ΔCt V2	Fold Change	Fold Regulation	*p*-Value
20a-5p	1.46 (1.05–1.87)	1.50 (1.33–1.66)	0.97	−1.03	0.945
23b-3p	1.69 (2.16–1.22)	1.10 (1.34–0.86)	1.51	1.51	0.203
29a-3p	0.12 (−0.44–0.67)	0.19 (0.11–0.49)	0.95	−1.05	0.868
92b-3p	5.70 (5.25–6.15)	6.05 (5.75–6.34)	0.78	−1.27	0.434
99a-5p	0.72 (1.14–0.29)	0.52 (0.68–0.36)	1.15	1.15	0.595
137-3p	19.33 (18.47–20.19)	18.64 (18.07–19.21)	1.61	1.61	0.252
143-3p	16.43 (14.35–18.51)	16.17 (14.13–18.22)	1.20	1.20	0.945
208a-3p	17.62 (16.53–18.71)	17.80 (16.76–18.84)	0.88	−1.13	0.945
302d-3p	19.50 (18.76–20.25)	18.64 (18.07–19.21)	1.82	1.82	0.203
379-3p	15.52 (13.88–17.16)	17.40 (16.23–18.57)	0.27	−3.68	0.203
543	15.68 (14.75–16.60)	16.58 (15.69–17.47)	0.54	−1.87	0.375
665	5.68 (5.04–6.31)	7.47 (6.89–8.05)	0.29	−3.46	**<0.001**

Data are expressed as estimated mean ΔCt, which was calculated as micro(mi)RNA of interest Ct—housekeeping miRNA Ct, and 95% confidence interval. MiRNA fold change between visits was calculated using the 2^(−ΔΔCt)^ method; when fold-change values were greater than one (upregulation), fold regulation was equal to the fold change; when fold-change values were less than one (downregulation), fold regulation was reported as the negative inverse of the fold change. Note that high values of ΔCt indicate lower miRNA expression, whereas low ΔCt values indicate higher miRNA expression. Significant *p*-values are denoted in bold.

**Table 10:** Panel of genes involved in neuropathic and inflammatory pain included in the customized PCR array.

Gene	Description	Gene	Description
*BDKRB1*	Bradykinin receptor B1	*NTRK1*	Neurotrophic tyrosine kinase, receptor, type 1
*BDNF*	Brain-derived neurotrophic factor	*OPRD1*	Opioid receptor, delta 1
*CALCA*	Calcitonin-related polypeptide alpha	*OPRK1*	Opioid receptor, kappa 1
*CCL2*	Chemokine (C-C motif) ligand 2	*OPRM1*	Opioid receptor, mu 1
*CD200*	CD200 molecule	*PENK1*	Proenkephalin 1
*CNR2*	Cannabinoid receptor 2 (macrophage)	*PTGS1*	Prostaglandin-endoperoxide synthase 1 (prostaglandin G/H synthase and cyclooxygenase)
*CX3CR1*	Chemokine (C-X3-C motif) receptor 1	*TAC1*	Tachykinin, precursor 1
*IL18*	Interleukin 18	*TACR1*	Tachykinin receptor 1
*IL1A*	Interleukin 1A	*TNF*	Tumor necrosis factor
*IL2*	Interleukin 2	*TRPA1*	Transient receptor potential caption channel 1
*IL6*	Interleukin 6	*TRPV1*	Transient receptor potential cation channel, subfamily V, member 1
*ITGAM*	Integrin, alpha M (complement component 3 receptor 3 subunit)	*TRPV3*	Transient receptor potential cation channel, subfamily V, member 3
*NGF*	Nerve growth factor		

## Data Availability

The original contributions presented in this study are included in the article and Supplementary Materials. Further inquiries can be directed to the corresponding author.

## Supplementary Materials

The following supporting information can be downloaded at: https://www.mdpi.com/article/10.3390/ijms26188918/s1.

## Abbreviations

The following abbreviations are used in this manuscript:

DED	Dry eye disease
IL	Interleukin
Ra	Receptor antagonist
MMP	Matrix metalloproteinase
MIP	Macrophage inflammatory protein
IFN	Interferon
miRNA	MicroRNA
TNF	Tumor necrosis factor
V1	First visit
V2	Follow-up visit
OSDI	Ocular Surface Disease Index
IVCM	In vivo confocal microscopy
EGF	Epidermal growth factor
MCP	Monocyte chemoattractant protein
GRO	Growth-related oncogene
SP	Substance P
RANTES	Regulated on activation, normal T cell expressed and secreted
NGF	Nerve growth factor
*BDKRB1*	Bradykinin receptor B1
*CALCA*	Calcitonin-related polypeptide alpha
*CCL2*	Chemokine (C-C motif) ligand 2
*ITGAM*	Integrin, alpha M (complement component 3 receptor 3 subunit)
*TAC1*	Tachykinin, precursor 1
*CD200*	CD200 molecule
*TACR1*	Tachykinin receptor 1
*CX3CR1*	Chemokine (C-X3-C motif) receptor 1
*BDNF*	Brain-derived neurotrophic factor
*NTRK1*	Neurotrophic tyrosine kinase, receptor, type 1
*PENK1*	Proenkephalin 1
*OPRM1*	Opioid receptor, mu 1
*OPRD1*	Opioid receptor, delta 1
*OPRK1*	Opioid receptor, kappa 1
*TRPV3*	Transient receptor potential cation channel, subfamily V, member 3
*TRPA1*	Transient receptor potential caption channel 1
*TRPV1*	Transient receptor potential cation channel, subfamily V, member 1
*CNR2*	Cannabinoid receptor 2 (macrophage)
*PTGS1*	Prostaglandin-endoperoxide synthase 1 (prostaglandin G/H synthase and cyclooxygenase)
CDES-Q	Change in Dry Eye Symptoms Questionnaire
TBUT	Fluorescein tear break-up time
CELab	Controlled Environment Laboratory
mSIDEQ	Modified Single-Item Dry Eye Questionnaire
Ct	Cycle threshold
*GAPDH*	Glyceraldehyde-3-phosphate dehydrogenase
