# Supplementary material for: Long-Term Clinical and Molecular Changes in Dry Eye Disease and Chronic Ocular Pain
Source: Int J Mol Sci. 2025 Sep 12;26(18):8918. doi: 10.3390/ijms26188918 (PMC12469960; doi:10.3390/ijms26188918)
Supplement: Supplementary file 1 [file ijms-26-08918-s001.zip › ijms-3787191-supplementary.pdf]

Supplementary Table S1. Lower limits of detection (pg/ml) for each standard curve.

	**V1**	**V2**
EGF	3.2	6.1
Fractalkine/CX3CL1	3.2	6.1
IL-1β	9.6	6.1
IL-1Ra	3.2	3.2
IL-2	3.2	6.1
IL-4	3.2	6.1
IL-6	6.14	15.3
IL-8/CXCL8	3.2	12.3
IL-9	3.2	6.1
IL-10	3.2	6.1
IL-17A	3.2	6.1
MCP-1/CCL2	9.6	18.3
MCP-3/CCL7	3.2	6.1
TNF-α	6.4	12.3
IFN-γ	3.2	6.1
GRO	3.2	6.1
MIP-1α/CCL3	3.2	6.1
MIP-1β/CCL4	3.2	6.1
NGF	3.2	6.1
RANTES/CCL5	3.2	6.1

V1: visit 1; V2: visit 2; EGF: epidermal growth factor; IL: interleukin; IL-1Ra: interleukin-1 receptor antagonist; MCP: monocyte chemoattractant protein; TNF: tumor necrosis factor; IFN: interferon; GRO: growth related oncogene; MIP: macrophage inflammatory protein; NGF: nerve growth factor; RANTES: Regulated on Activation Normal T Cell Expressed and Secreted.
